# Downregulation of *ALAS1* by nicarbazin treatment underlies the reduced synthesis of protoporphyrin IX in shell gland of laying hens

**DOI:** 10.1038/s41598-017-06527-y

**Published:** 2017-07-24

**Authors:** Sami Samiullah, Juliet Roberts, Shu-Biao Wu

**Affiliations:** Animal Science, School of Environmental and Rural Science, University of New England, Armidale, New South Wales 2351 Australia

## Abstract

Shell colour is an important trait for eggs and an understanding of pigment deposition will assist potential management of egg shell colour loss. We demonstrated that nicarbazin feeding down-regulated *ALAS1* and reduced protoporphyrin IX (PP IX) in both shell gland and eggshell, indicating the role of nicarbazin in inhibiting the synthesis of PP IX. Additionally, the expression levels of the genes did not show sequential upregulation in the same order of diurnal time-points (TP) during egg formation. The gene *SLC25A38*, responsible for transporting glycine from cytoplasm to mitochondria, and the gene *ALAS1*, encoding rate-limiting enzyme (delta-aminolevulinic acid synthase 1), had higher expression at 15 hr, as compared with 2, 5 and 23.5 hrs postoviposition. Interestingly, *ABCB6*, a gene encoding an enzyme responsible for transporting coproporphyrinogen III, showed higher expression level at 2 and 5 hrs. However, the expression of *CPOX* that converts coproporphyrinogen III to protoporphyrinogen III, and *ABCG2* that transports PP IX out from mitochondria did not alter. Nevertheless, mitochondrial count per cell did not show consistent change in response to time-points postoviposition and nicarbazin feeding. The information obtained in the study sheds light on how nicarbazin disrupts the synthesis of PP IX.

## Introduction

The oviduct in laying hens is divided into five distinguishable regions: infundibulum (10 cm), magnum (35 cm), isthmus (8 cm), shell gland (10 cm) and vagina (4 cm)^1, 2^. The formation of the egg takes about 24 hrs during which it sequentially passes through various parts of the oviduct. The release of the next ovum is precisely synchronized, occurring an average of 24 minutes post-oviposition under a 24 hr light-dark cycle^3^. The ovum remains in the infundibulum for about 0.5 hr, in the magnum for about 3 hrs, in the isthmus for 1–2 hrs, and in the shell gland for about 18–20 hrs^2, 4, 5^. The eggshell comprises both organic and inorganic components that are synthesised in the isthmus and shell gland^6–9^. The eggshell is composed of shell membranes, mammillary layer, palisade layer, surface crystal layer and cuticle^10, 11^. The brown eggshell colour in laying hens is mainly due to the deposition of a pigment known as protoporphyrin IX (PP IX) during eggshell formation in the shell gland. The presence of PP IX in various shell layers has been reported^12–14^ although most of the pigment is deposited into the outmost layers of the palisade layer (80–87%) with a smaller amount in the cuticle (13–20%)^15^. Shell deposition increases regularly 12–23 hrs post-oviposition and reaches a plateau 1.5 hrs before oviposition^16^, which coincides with the deposition of cuticle (1–1.5 hrs)^17^. PP IX deposition increases greatly during the last 20–24 hrs post-oviposition^18^.

It is assumed that regular egg formation may change the energy metabolism of key organs such as the oviduct, liver and adipose tissue. At the cellular level, alterations of nutrient and energy requirements coincide with changes in the mitochondria, the main site of production of ATP in cells^19^. The mitochondrial genome has multiple copies per cell and the number of mitochondria varies depending on energy demands of a cell, the age and sex of an organism, the organ and patho-physiological conditions^20–22^. Certain drugs are nucleoside analogs that block the progression of polymerase γ^23^, which leads to the inhibition of mitochondrial biogenesis. The mitochondrial DNA (mtDNA) copies decrease with age, as shown in mice and humans^24^. However, mtDNA copies increased from early to late lactation in the mammillary glands of cows^25^. The mtDNA replication is independent of the cell cycle and is controlled by the nuclear DNA encoded polymerase γ^26, 27^ and mitochondrial transcription factor A^28^.

Chicken (*Gallus gallus*) mtDNA is circular in shape with about 16758 bp, containing 37 identified genes encoding 13 polypeptides, 2 rRNAs, 22 tRNAs and 1 non coding control D-loop region^29, 30^. This small genome has been shown to contain around 15% of repetitive DNA organized as short tandem repeats, such as telomeric and centromeric tandem repeats^31^. Determination of mtDNA content is important for understanding many cellular processes^32^. An essential component of mitochondrial biogenesis is the regulation of mitochondrial morphology and number within healthy cells with a dynamic balance of fission and fusion events. The number of mtDNA copies is highly dynamic and regulates in a cell-specific manner by mechanisms that are not completely understood^33, 34^.

In the epithelial cells of the chicken shell gland, mitochondria are of particular interest, owing to the high demand for energy used in the biogenesis of the eggshell. Mitochondrial biogenesis in these cells also allows them to meet changing energy loads for production of various components of the eggshell, including eggshell pigment. Nicarbazin is one of the various factors that has been shown to cause reduced synthesis and/or deposition of protoporphyrin into eggshells, when fed to brown-egg laying hens at recommended dosages (50–125 mg/kg of feed)^35, 36^. Nicarbazin produces reversible pharmacological effects by causing decolouration of brown egg pigment, which is dosage dependent^37, 38^. Thus, this model can be easily reproduced in studies to investigate effects of nicarbazin on eggshell formation in laying hens. Understanding the molecular basis for the effect of nicarbazin may also provide an understanding of the mechanisms by which shell colour decreases in response to other factors which have been less well-defined.

**Figure 1:**
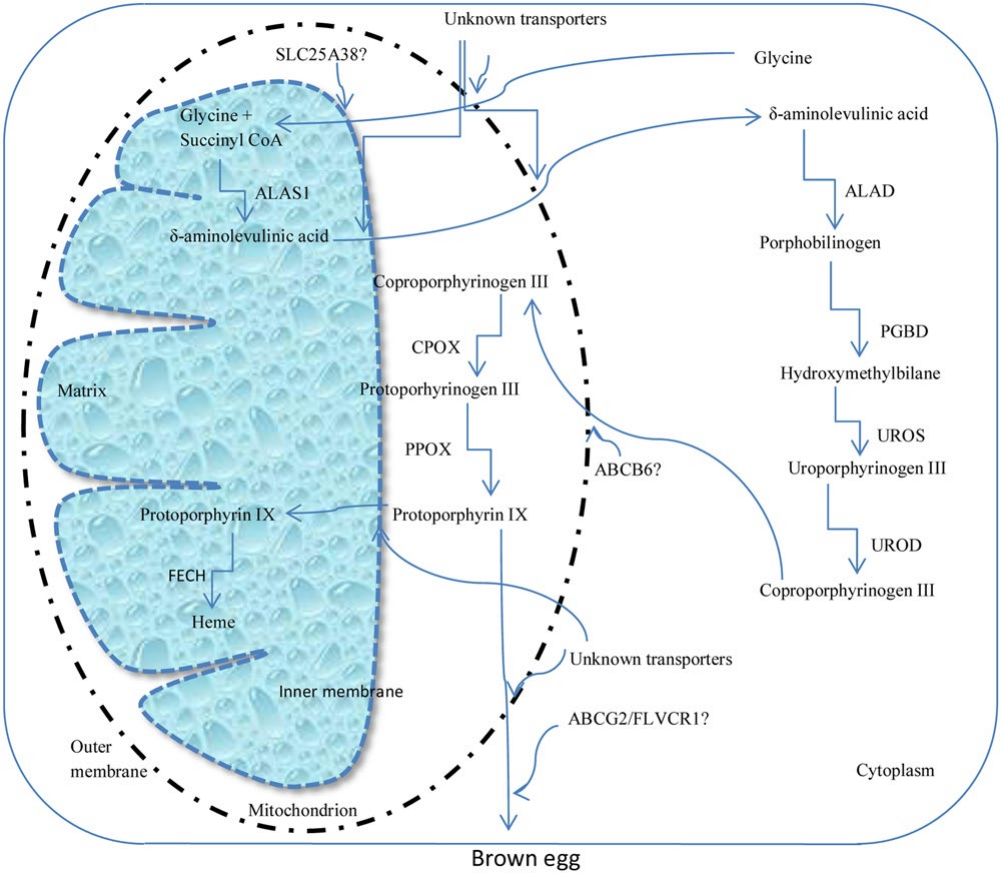
Schematic representation of protoporphyrin IX biosynthetic pathway in the shell gland of brown egg laying hens. Glycine is transported from the cytosol to the matrix of mitochondria through the possible transporter SLC25A38. Succinyl coenzyme A and glycine are oxidized by aminolevulinic acid synthase 1 (ALAS1) to δ-aminolevulinic acid, which is transported back to the cytosol through unknown transporter/s. The δ-aminolevulinic acid is converted into porphobilinogen through the action of aminolevulinic acid dehydratase (ALAD). The porphobilinogen is further reduced into hydroxymethylbilane through porphobilinogen deaminase (PGBD). In the next two steps, uroporphyrinogen III and coproporphyrinogen III are synthesized through the actions of uroporphyrinogen III synthase (UROS) and uroporphyrinogen decarboxylase (UROD), respectively. The coproporphyrinogen III is transported back to mitochondria and is converted to protoporhyrinogen III through the action of coproporphyrinogen oxidase (CPOX). The protoporphyrinogen III is converted into protoporphyrin IX through protoporphyrinogen oxidase (PPOX) that deposits into eggshell. It is not known, how the protoporphyrin IX is inhibited from being converted into heme, a part of haemoglobin.

The biosynthetic pathway of PP IX is well established; however, the origin of its precursors and the mechanism of deposition are still not known. The pigment is believed to be synthesized in the mucosal epithelial cells of the shell gland where the whole process takes place in the mitochondria and cytoplasm with the involvement of various enzymes. Briefly, as illustrated in Fig. 1, the solute carrier family 25, member 38 (*SLC25A38*) gene, functions to facilitate delta-aminolevulinic acid production by transporting glycine into the mitochondrial matrix from the cytosol^39^. The delta-aminolevulinic acid synthase 1 (*ALAS1*) gene encodes a rate limiting non-erythroid enzyme that catalyses the reaction of succinyl coenzyme A with glycine to form delta-aminolevulinic acid within the mitochondrial matrix^40, 41^. Cell surface ATP-binding cassette (ABC) transporters serve to efflux a variety of compounds^42^ and are also involved in the transport of substances across mitochondrial membranes^43^. The gene *ABCB6* is part of the ATP-binding cassette family and is located on the mitochondrial membrane^44^. This gene encodes a transporter that has been demonstrated to play a role in transporting coproporphyrinogen III back to the mitochondria from the cytosol^45^. The coproporphyrinogen oxidase (*CPOX*) gene encodes an enzyme that converts coproporphyrinogen III into protoporphyrinogen III and this gene is located in the mitochondria^46^. *ABCG2* is also part of the ATP-binding cassette family but plays a role in the export of PP IX out of the cell^47, 48^. The feline leukemia virus subgroup C cellular receptor 1 (*FLVCR1*) gene is located on the cell membrane that encodes an enzyme that transports heme/PP IX out of the cell^45^. This gene is highly conserved throughout evolution with orthologs present in plants, bacteria and animals, suggesting its importance in cell biology^49^. The ferrochelatase gene (*FECH*) encodes the enzyme that catalyses the final step in converting PP IX into heme^50, 51^.

Based on the active involvement of the avian shell gland in the production of various components of eggshells, we hypothesized that expression levels of the genes involved in the synthesis of PP IX may change according to their roles in the synthesis procedure following a temporal manner and possibly mitochondrial numbers in the cell of shell gland vary with different stages of eggshell formation. To test this hypothesis, we studied the possible variations in the expression of relevant genes and mitochondrial count per cell at different stages of egg/eggshell formation (time-points). We also hypothesized, on the basis of the effects of nicarbazin on production of PP IX, that nicarbazin may disrupt the mechanism involved in PP IX production by either regulating the genes involved in its production or affecting the mitochondrial numbers in the cells, resulting in lower production of PP IX appearing in eggshells of nicarbazin fed brown-egg laying hens. Nicarbazin was used as a model as it has been shown to produce reversible loss of brown eggshell colour^36, 37^. Therefore, Experiment 1 was performed to study the expression of genes involved in the synthesis of PP IX at different stages of egg/eggshell formation. In Experiment 2, the mechanism by which nicarbazin disrupts PP IX synthesis at different time-points of eggshell formation was studied. In both the experiments, the mitochondrial count per cell was quantified at different stages of egg/eggshell formation and during nicarbazin treatment, to establish any relationship between mitochondrial copy number and PP IX synthesis. The PP IX level was measured in shell gland tissue and eggshell to understand the effect of expression levels of genes on the synthesis and deposition of PP IX. Thus, the main objective of the study was to investigate the expression levels of genes involved in the synthesis of PP IX, as well as mitochondrial count, at different stages of egg/eggshell formation and during nicarbazin treatment in brown-egg laying hens.

## Results

### Mitochondrial count per cell and PP IX synthesis genes

**Figure 2:**
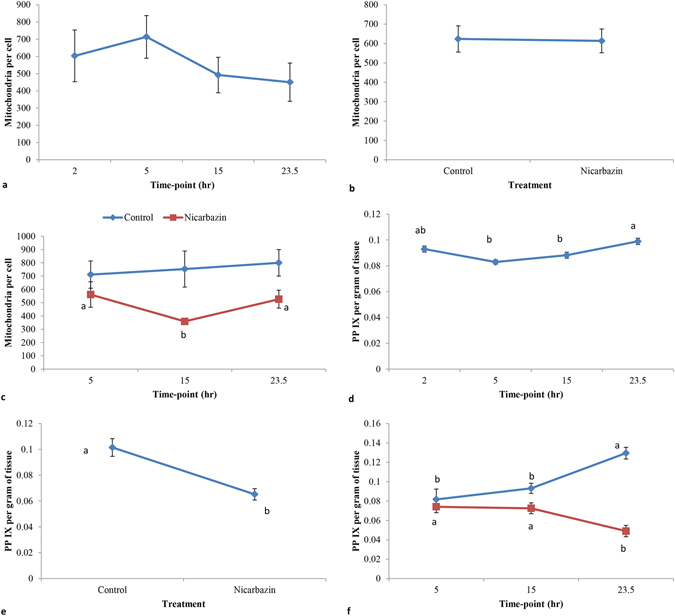
Mitochondria per cell and PP IX per gram of shell gland tissue affected by time-points of eggshell formation and nicarbazin treatment. (**a**) Mitochondria per cell in the shell gland tissue of laying hens affected by four different time-points of eggshell formation. (**b**) Mitochondria per cell in the shell gland tissue between control and nicarbazin treated laying hens. (**c**) Mitochondria per cell affected by interaction between three different time-points and nicarbazin treatment. (**d**) PP IX (in nMoles) per gram of shell gland tissue affected by four different time-points of eggshell formation. (**e**) PP IX (in nMoles) per gram of shell gland tissue between the control and nicarbazin treated hens. (**f**) PP IX (in nMoles) per gram of shell gland tissue affected by three different time-points and nicarbazin treatment. Values are mean and bars represent standard error. Different superscripts ^(**a**,**b**)^ show significant difference.

In Experiment 1, the mean mitochondrial count per cell in the shell gland tissue was not significantly different (P > 0.05) among the four different stages of egg/eggshell formation (time-points) (Fig. 2a). However, the expression levels of the candidate genes were significantly affected (P < 0.05) by the time-points except for the *CPOX* and the *ABCG2* (Table 1). The expression level of *SLC25A38* gene was significantly higher at 15 hr compared with the 2, 5 and 23.5 hrs post-oviposition times. The expression level of *ALAS1* gene was significantly higher at 5 and 15 hrs compared with 2 and 23.5 hrs post-oviposition. The expression level of *ABCB6* gene was significantly higher at 2, 5 and 15 hrs post-oviposition compared with 23.5 hr. The expression levels of *FECH* and *FLVCR1* genes were significantly higher at 23.5 hr compared with the other post-oviposition times.

**Table 1:** Relative expression levels of candidate target genes involved in the synthesis of protoporphyrin IX and/or heme at different time-points of eggshell formation in the shell gland of laying hens.

Gene	Time-point (hr)				P value
	2	5	15	23.5	
*SLC25A38*	0.87 ± 0.12^b^	0.84 ± 0.12^b^	1.74 ± 0.12^a^	0.94 ± 0.13^b^	0.0019
*ALAS1*	0.79 ± 0.05^c^	1.32 ± 0.09^b^	1.69 ± 0.10^a^	0.59 ± 0.05^c^	<0.0001
*ABCB6*	1.23 ± 0.05^a^	1.25 ± 0.13^a^	1.06 ± 0.11^a^	0.67 ± 0.11^b^	0.0047
*CPOX*	0.96 ± 0.05	1.03 ± 0.04	1.11 ± 0.09	0.95 ± 0.06	0.3727
*ABCG2*	1.04 ± 0.08	1.15 ± 0.08	0.87 ± 0.05	1.01 ± 0.06	0.0936
*FECH*	0.88 ± 0.04^b^	0.85 ± 0.05^b^	0.80 ± 0.05^b^	1.72 ± 0.04^a^	<0.0001
*FLVCR1*	0.97 ± 0.04^b^	0.85 ± 0.05^b^	1.03 ± 0.07^ab^	1.23 ± 0.12^a^	0.0255

Values are the mean of normalized relative quantities (NRQ) ± standard error. Relative quantities for individual gene are scaled to the average across all unknown samples per target gene. Different superscript letters ^a,b,c^ across a row denote significantly different results.

In Experiment 2, no main effect of either time-point or nicarbazin treatment was observed on the mitochondrial count (Fig. 2b). There was significant interaction (P < 0.05) of time-points and nicarbazin treatment for mitochondrial count at 15 hr time-point (Fig. 2c). The nicarbazin decreased mitochondrial count only at 15 hr post-oviposition but not at other time-points. The expression levels of all the genes except for *CPOX* were significantly affected by time-points, both in the control and nicarbazin treatment groups (Table 2). The expression level of *SLC25A38* was significantly higher at 15 hr both in the control and nicarbazin treatment groups (Table 2). *ALAS1* expression level was significantly higher at 15 hr compared with the 5 and 23.5 hrs in the control group. However, in the nicarbazin treatment groups, *ALAS1* expression level showed no significant difference between 5 and 15 hrs. *ABCG2* expression level was significantly higher at 5 hr compared with both the 15 and 23.5 hrs time-points in the control and in the nicarbazin treatment groups. The higher expression level of *FECH* was the same in the control and nicarbazin treatment groups, occurring at 23.5 hr. In both the groups, the *FLVCR1* expression level was significantly higher at 15 hr time-point. There was no significant interaction between time-points and nicarbazin treatment for any of the genes expression levels investigated (Table 2). The expression levels of all seven genes studied were not significantly different (P > 0.05) between the control and nicarbazin treatment hens except for *ALAS1* (Table 2). In the control group, the mean relative expression levels (±S.E.) of *ALAS1* at 5, 15 and 23.5 hrs time-points were 1.34 ± 0.06, 1.66 ± 0.07 and 0.60 ± 0.03, respectively. In the nicarbazin treatment group, the mean relative expression levels (±S.E.) of *ALAS1* at 5, 15 and 23.5 hrs time-points were 1.21 ± 0.09, 1.27 ± 0.06 and 0.50 ± 0.01, respectively.

**Table 2:** Relative expression levels of candidate target genes involved in the synthesis of protoporphyrin IX and/or heme at different time-points of eggshell formation in the shell gland of laying hens treated with nicarbazin.

Gene	Time-point (hr)			Treatment		P value		
	5	15	23.5	Control	Nicarbazin	TP	N	TP*N
*SLC25A38*	0.75 ± 0.03^b^	1.71 ± 0.07^a^	0.82 ± 0.07^b^	1.04 ± 0.11	1.14 ± 0.13	<0.0001	0.1567	0.3481
*ALAS1*	1.28 ± 0.09^b^	1.47 ± 0.08^a^	0.55 ± 0.02^c^	1.20 ± 0.09^a^	0.99 ± 0.06^b^	<0.0001	0.0104	0.2148
*ABCB6*	1.32 ± 0.08^a^	1.02 ± 0.06^ab^	0.80 ± 0.07^b^	1.06 ± 0.06	1.03 ± 0.08	0.0002	0.6805	0.7290
*CPOX*	1.07 ± 0.04	1.03 ± 0.02	0.93 ± 0.04	1.02 ± 0.04	1.00 ± 0.03	0.0700	0.7199	0.7765
*ABCG2*	1.34 ± 0.09^a^	0.87 ± 0.04^b^	0.91 ± 0.08^b^	1.04 ± 0.05	1.05 ± 0.10	0.0002	0.9335	0.1560
*FECH*	0.78 ± 0.02^b^	0.72 ± 0.03^b^	1.80 ± 0.05^a^	1.07 ± 0.13	1.13 ± 0.13	<0.0001	0.1467	0.7750
*FLVCR1*	0.79 ± 0.02^c^	1.26 ± 0.07^a^	1.05 ± 0.09^b^	1.07 ± 0.07	1.00 ± 0.07	0.0002	0.3850	0.1792

The time-point and nicarbazin treatment columns show main effects on the expression levels of the genes. Values are the mean of normalized relative quantities (NRQ) ± standard error. Relative quantities for individual gene are scaled to the average across all unknown samples per target gene. Within each treatment, different superscript letters ^a,b,c^ across a row denote significantly different results. TP is time-point, while N denotes nicarbazin treatment.

### Measurement of PP IX in shell gland tissue

In Experiment 1, the mean values of PP IX measured per gram of shell gland tissue were significantly affected (P < 0.05) by time-point (Fig. 2d). The PP IX level in the shell gland was at the lowest when the egg was not in the shell gland at 5 hr post-oviposition then gradually increased to the highest at 23.5 hr (Fig. 2d).

In Experiment 2, there was a significantly lower amount of PP IX per gram of shell gland tissue in the nicarbazin treated compared with the control groups (Fig. 2e). A significant interaction between time-point and treatment showed that, in the control groups, the amount of PP IX significantly increased at the 23.5 hr post-oviposition time whereas, in the nicarbazin treatment groups, the amount of PP IX declined (Fig. 2f).

### Measurement of PP IX in eggshell

**Figure 3:**
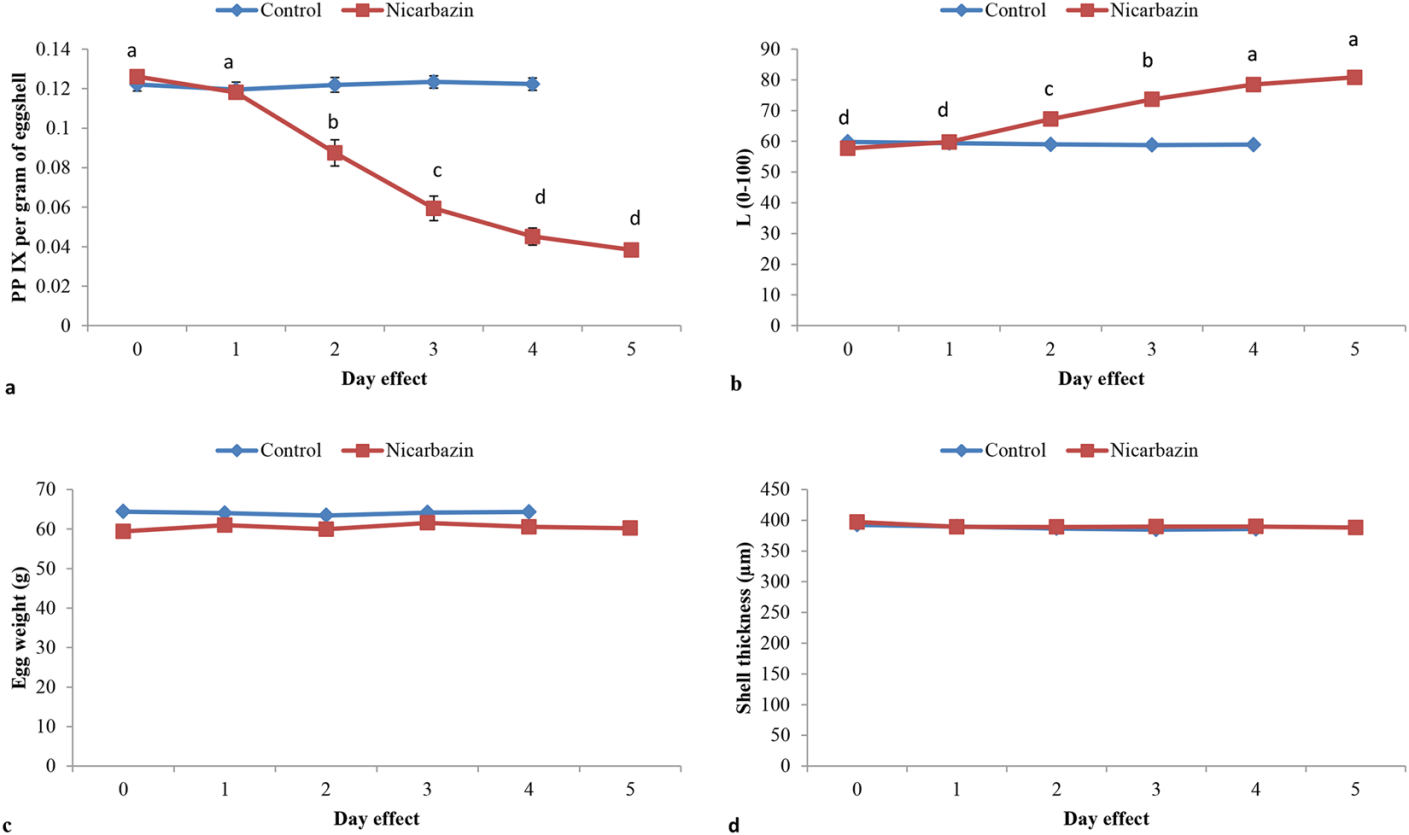
Eggshell quality variables measured in control and nicarbazin treated laying hens affected by day effect. (**a**) PP IX per gram of eggshell (nMoles) between the control (P = 0.9512) and nicarbazin (P < 0.0001) treated hens. (**b**) L* values for eggshells between the control (P = 0.7026) and nicarbazin (P < 0.0001) treated hens. (**c**) Egg weight between the control (P = 0.9683) and nicarbazin (P = 0.6414) treated hens. (**d**) Shell thickness of eggshells between the control (P = 0.8345) and nicarbazin (P = 0.8600) treated hens. Values are mean and bars represent standard error. Different superscripts ^(a, b, c, d)^ show significant difference.

In Experiment 2, in the eggshells from the control groups, the amount of PP IX and L* values remained constant whereas, in the nicarbazin treatment groups, PP IX decreased linearly with increasing duration of administration (day effect) of the drug, as indicated by the lower amount of PP IX and higher values of L* (Fig. 3a and b). Egg weight and shell thickness were not significantly different between the control and nicarbazin treated groups (Fig. 3c and d).

## Discussion

The first step in the initiation of PP IX biosynthetic pathway is the transportation of glycine from cell cytoplasm to mitochondria. *SLC25A38* has been shown to be the transporter for glycine, which is located on mitochondrial inner membrane^39^. A significantly higher expression of *SLC25A38* at the 15 hr time-point observed in the current study may indicate that, during this stage, the synthesis of PP IX has already been initiated for the programmed deposition into the shell of the egg to be laid on that day or even the next day. This is further confirmed by the significantly higher expression level of the rate limiting enzyme ALAS1 at the same time-point of eggshell formation. The genes that encode proteins involved in the subsequent steps of the PP IX synthesis did not follow the same temporal sequence on the day. For example, *ABCB6* was expressed at significantly higher level at the 5 hr time-point while its role in the PP IX synthetic pathway is at later stage. In the PP IX biosynthetic pathway, it is not clear how the genes involved are co-regulated as the transporters or enzymes. The proteins involved come from different sources and may not necessarily be synthesized at the same level, in the same location of the cell, and in the same duration for their functions.

In the current study, the sampling time-points (time post-oviposition) 2 and 5 hrs were prior to the entry of the forming egg into the shell gland at approximately 5–6 hrs postoviposition. At the 15 hr time-point, the eggshell is undergoing rapid growth of the palisade columns^52^. By the 23.5 hr time-point, mineralisation will have ceased and the cuticle will be forming. Most PP IX is deposited from 21 hours postoviposition to oviposition^18^. Therefore the genes involved in PP IX synthesis may be up-regulated well before the formation of the eggshell. Despite extensive studies on the macromolecules involved in shell formation, the spatio-temporal expression of the genes involved in the sequential synthesis of various layers of the eggshell and PP IX is not clearly established.

In the current study, the expression level of *CPOX* was not significantly changed between the different time-points. The protein encoded by *CPOX* gene is the enzyme that catalyses the sixth step of the PP IX/heme biosynthetic pathway^46^. The encoded enzyme is soluble and found in the intermembrane space of mitochondria^53^. This enzyme catalyses the stepwise oxidative decarboxylation of coproporphyrinogen III to protoporphyrinogen IX, a precursor of PP IX^46, 54^. Interestingly, Zheng *et al*.^55^, observed a correlation between the level of *CPOX* and the level of PP IX synthesis in brown-egg laying hens. This may suggest that sufficient *CPOX* RNA expression is maintained at all the time-points when the data were collected in the current study for the synthesis of PP IX.

The different expression levels of most genes, with the exception of *ABCG2* and *CPOX*, in relation to different time-points, indicate that genes involved in PP IX synthesis expressed differently during eggshell formation. As mentioned earlier, it is not clear how the regulation of various genes is synchronized in a multi-step biosynthetic pathway of PP IX. Therefore, the dynamics of the expression of these genes including *CPOX* needs to be investigated in a temporal and spatial manner. Based on the effects of time-points on the expression levels of genes, it is assumed that these genes are transcribed and transported at different time-points; however, functionally coordinated for the synthesis of PP IX. Nevertheless, the differential expression of the genes suggests that these genes are essential in the synthesis of PP IX; thus the regulation of the genes may affect the deposition of the pigment into the eggshell.

As mentioned previously, *ALAS1* gene encodes an enzyme that catalyses the rate-limiting step in PP IX/heme biosynthetic pathway^40^. The enzyme encoded by *ALAS1* gene is delta-aminolevulinic acid synthase 1; a separate gene encodes a form of the enzyme (*ALAS2*) that is specific for erythroid tissue^56–58^. The level of the mature encoded protein is regulated by heme: high levels of heme down-regulate the mature enzyme in mitochondria while low heme levels up-regulate^56, 59, 60^. However, in the shell gland tissue of brown-egg laying hens, the PP IX rather than heme is the final product that deposits into various layers of eggshell. The role of the *FECH* gene in the PP IX biosynthetic pathway, the gene that encodes the enzyme that converts PP IX into heme, is still not clear. In our study, the expression level of *FECH* gene was significantly higher at 23.5 hr, but this could not be aligned in a temporal order with the expression of the other genes studied. Based on studies performed in mammalian^61^, bacteria^62^ and avian^60^ tissues, a negative feedback mechanism exists and the formed heme in turn acts as a regulator of the PP IX biosynthetic pathway. If *FECH* has an active role in the PP IX pathway in the chicken shell gland tissue, the question arises as to how the heme formed in the epithelial cells of the shell gland is transported back to the blood to become a part of the haemoglobin molecule or for degradation. Also, there should be more heme in the shell gland cells rather than PP IX.

The higher expression level of *FLVCR1* at 15 hr can be linked with the expression levels of other genes, also suggesting the initiation of PP IX synthesis around this time-point. A significantly higher expression of the *FLVCR1* gene in the shell gland of laying hens with darker brown eggshells has been reported^63^; however, there are no published reports confirming whether the *FLVCR1* gene is involved in exporting and/or depositing PP IX onto eggshells in the shell gland of laying hens. Further study on its functional role in the PP IX deposition into eggshell is needed.

In the comparison of the expression levels of gene expression data between the control and nicarbazin treatment groups, *ALAS1* was the only gene affected by nicarbazin. It was observed that nicarbazin reduced *ALAS1* expression across all the time-points and thus a lower amount of PP IX was produced in the shell gland. However, the lower expression level of *ALAS1* did not affect the expression levels of the remaining genes studied, as their expression levels were similar between the control and nicarbazin treated hens indicating that no feedback mechanisms exist to such extent for the regulation of the genes involved in the PP IX synthesis pathway. The current study suggests that nicarbazin disrupts PP IX synthesis by affecting the expression level of the *ALAS1* gene. Nicarbazin is a chemically produced drug composed as a complex of an equimolar amount of 4,40-dinitrocarbanilide (DNC) and 2-hydroxy-4,6-dimethylpyrimidine (HDP). The DNC moiety has been shown to inhibit PP IX synthesis^64^, but how the DNC moiety inhibits the expression of *ALAS1* gene is not clear. To the best of our knowledge, this is the first observation that nicarbazin regulates PP IX production through regulating *ALAS1* gene expression either directly or indirectly.

The PP IX biosynthetic pathway is well established; however, the origin of precursors and the cells involved in its synthesis in the shell gland of brown-egg laying hens are not yet fully understood (reviewed in Samiullah *et al*.)^65^. Not all of the enzymes involved in the PP IX/heme biosynthetic pathway are the same between erythroid and non-erythroid cells and differences at their mRNA levels have been reported for ALAS, ALAD and PBGD (reviewed in Ponka)^59^. Similarly, in a mouse model, it has been shown that *CPOX* gene is differentially regulated in erythroid and non-erythroid cells^46^. However, it is not clear whether there is different erythroid and non-erythroid specific regulation of other genes involved in the PP IX/heme biosynthetic pathway. The current study is a preliminary investigation highlighting the role of nicarbazin in disrupting the PP IX biosynthetic pathway without conclusively demonstrating the site of PP IX production in the shell gland tissue of brown-egg laying hens. Due to the lack of conclusive information about the location of genes involved in the pathway and about how nicarbazin affects the biosynthetic pathway of PP IX, we decided to process whole pieces of shell gland tissue for the gene expression study, mitochondrial quantification and quantification of PP IX from the tissue. Nevertheless, the data presented in the current study have shown that differentially expressed *ALAS1* gene was detectable in response to the nicarbazin treatment. Thus, investigation into the tissue including epithelia and muscle appears to be effective in determining gene expression in the PP IX biosynthetic pathway.

The fact that there was no difference in egg weight and shell thickness between the control and nicarbazin treatment groups also indicates that nicarbazin does not affect genes involved in eggshell formation including the transport of calcium and phosphorus across the shell gland cells. However, these variables were not measured directly, and the nicarbazin was fed only for a short period. It would be valuable to study the long term effects of nicarbazin on gene expression levels associated with shell matrix formation, as earlier studies have shown a significant deterioration in egg quality when feeding nicarbazin to hens for longer periods^66, 67^.

Overall, the expression levels of genes involved in the production of PP IX differed in relation to the stages of eggshell formation. Feeding nicarbazin to brown-egg laying hens inhibited the expression level of *ALAS1*, resulting in lower production of PP IX for incorporation into the eggshells. The nicarbazin model could assist in understanding the mechanism of reduced synthesis and/or deposition of PP IX into eggshells due to different factors; however, the mechanism of action of individual factors in lower synthesis of PP IX may not necessarily be the same as for nicarbazin. This study confirmed that mitochondrial count per cell in the shell gland tissue does not vary significantly with different stages of egg/eggshell formation. Nevertheless, further investigation is required to understand the turnover of mitochondria in tissues such as the oviduct of laying hens. This study provides information on the interaction of nicarbazin drug with *ALAS1* gene by reducing its expression in the shell gland of brown-egg laying hens. Furthermore, this study may be used as a base for future studies in which the effect of nicarbazin and other factors on the mechanism of PP IX synthesis can be more broadly investigated.

## Methods

### Ethics statement and rearing of laying hens

The following experimental protocol was approved by the Animal Ethics Committee (Authority No.: AEC15-022) of the University of New England, Armidale, NSW, 2351, Australia. The protocol was carried out in accordance with the guidelines specified in the Australian Code for the Care and Use of Animals for Scientific Purposes 8^th^ edition 2013. Briefly, Hy-Line brown-egg laying hens were reared in individual cages with food and water provided *ad libitum*. The food provided was premium top layer mash (Barastock, Australia), containing crude protein (minimum) 16.5%, crude fat (minimum) 2.5%, crude fibre (maximum) 6%, salt (maximum added) 0.3%, copper (added) 8 mg/kg, selenium (added) 0.3 mg/kg and calcium (minimum) 3.6%. At the time of the experiment, hens were on a 16:8 hour light:dark photoperiod.

### Experiment 1: Effect of time-points on mitochondrial count and expression of protoporphyrin IX synthesis genes

The laying hen rearing shed was equipped with a video surveillance camera to precisely record the oviposition time of the hens selected for shell gland tissue collection. Eggs were collected and shell colour (L*) was measured with a spectrophotometer (Konica Minolta CM-2600d Ramsey, NJ, USA) using the L*a*b* colour space system. The L* measurements represent the intensity of brown eggshell colour; the higher the value, the lighter the colour of the eggshell. Egg weight was measured using an analytical balance, and the eggs were further processed for protoporphyrin IX (PP IX) quantification following the method of Samiullah and Roberts^15^. The post-oviposition times (time-points) were 2, 5, 15 and 23.5 hrs for the four groups of hens selected. Of 63 hens (36–37 weeks old), 20 hens were selected and divided in such a way that the mean values of the variables measured were not significantly different among the groups (Table 3). As the quantity of PP IX in eggshell may vary from hen to hen, the main purpose of recording these egg quality measurements was to ensure that hens were divided uniformly on the basis of shell weight and colour into the respective groups prior to the experiment.

**Table 3:** Mean values (±S.E.) of egg quality variables measured before dividing hens into different groups.

Variable	Time-point (hr)				P value
	2	5	15	23.5	
Egg weight (g)	62.8 ± 3.50	63.9 ± 2.30	62.9 ± 1.67	65.6 ± 1.76	0.8226
L* (0–100)	56.2 ± 0.98	54.4 ± 0.97	54.2 ± 0.88	54.4 ± 0.27	0.2984
PP IX in whole eggshell	0.14 ± 0.02	0.14 ± 0.03	0.14 ± 0.02	0.14 ± 0.02	0.9896
PP IX in calcareous shell	0.12 ± 0.02	0.11 ± 0.03	0.11 ± 0.02	0.11 ± 0.04	0.7170
PP IX in cuticle	0.02 ± 0.06	0.03 ± 0.04	0.03 ± 0.04	0.03 ± 0.04	0.6708

Protoporphyrin IX (PP IX) was measured in nMoles per gram of shell with and without cuticle and in cuticle alone. The eggs were collected before dividing the experimental hens into various groups. On the basis of eggshell variables, hens were divided into groups in such a way that the variables were not significantly different among groups.

### Experiment 2: Effect of time-points and nicarbazin treatment on mitochondrial count and expression of protoporphyrin IX synthesis genes

The rearing conditions for the hens were the same as described in Experiment 1. Of 43 hens (42–45 weeks old), 30 hens were selected on the basis of L*, egg weight and PP IX measurements of eggshell and divided equally into two groups, control and nicarbazin treatment (Table 4). Based on the results from Experiment 1, it was found that the gene expression values of time-points 2 and 5 hrs were not significantly different for most of the genes. Therefore, time-point 2 hr was omitted in Experiment 2. Time-point 5 hr was chosen because, at this time, the egg is either in the distal magnum and/or isthmus and preparing to move into the shell gland in the subsequent hour or so. Next, each group was subdivided into three groups by post-oviposition time (5, 15 and 23.5 hrs). Therefore, the experimental design was in a 2 × 3 factorial arrangement. For the control groups, the feed was the same as described earlier. For the nicarbazin treatment groups, each group was offered the same feed supplemented with nicarbazin powder (M.W. 426.38; Sigma Aldrich, Australia) at a rate of 100 mg/kg of feed. In a pilot experiment, it was determined that feeding nicarbazin at a rate of 100 mg/kg of feed was sufficient to cause complete discoloration of brown eggshell colour without causing any adverse effects on egg formation and feed intake. From the control groups, eggs were collected for analysis at least five days before processing the hens for shell gland tissue collection. From the nicarbazin treatment groups, eggs were collected for analysis at least one day before treatment and continued until the maximum loss in shell colour (>20%) was achieved. Individual hens were processed for shell gland tissue collection at specific time-points postoviposition.

**Table 4:** Mean values (±S.E.) of egg quality variables measured before dividing hens into different groups (time-points and nicarbazin treatment).

Variable	Group							
	Control (15 hens)				Nicarbazin (15 hens)			
	Time-point (hr)			P value	Time-point (hr)			P value
	5	15	23.5		5	15	23.5	
Egg weight (g)	63.7 ± 1.76	63.3 ± 1.60	63.8 ± 1.60	0.7070	59.6 ± 1.70	59.9 ± 1.41	62.6 ± 1.20	0.2102
L* (0–100)	58.7 ± 0.60	59.5 ± 0.90	59.4 ± 0.64	0.6820	58.5 ± 0.68	58.9 ± 0.55	59.1 ± 0.52	0.7652
PP IX	0.12 ± 0.05	0.13 ± 0.06	0.12 ± 0.06	0.7055	0.12 ± 0.05	0.13 ± 0.03	0.13 ± 0.03	0.4532

Protoporphyrin IX (PP IX) was measured in nMoles per gram of whole eggshell. The eggs were collected before dividing the experimental hens into various groups. On the basis of eggshell variables, hens were divided into groups in such a way that the variables were not significantly different among groups.

### Shell gland tissue collection

For both experiments, individual hen oviposition times were recorded, and each hen was euthanised at a specific post-oviposition time (2, 5, 15, 23.5 hrs and 5, 15, 23.5 hrs for Experiments 1 and 2, respectively). Hens were humanly euthanized with CO_2_ gas and the shell gland was aseptically collected through the abdominal incision. The shell gland was opened from the anterior-ventral side and an approximately 500 mg tissue was cut from the centre of the shell gland and transferred to RNALater (Sigma Aldrich, Australia) in 2 mL Eppendorf tube. The samples were stored at −20 °C and were processed for RNA/DNA extraction within one day of collection.

### Total RNA and DNA extraction

Total RNA and DNA were extracted from a whole piece of shell gland tissue (all tissue layers) that had been stored in RNALater at −20 °C. The TRIsure (Bioline, Australia) protocol was followed with slight modifications. Briefly, approximately 50 mg of tissue (wet weight) was homogenized in 1 mL of TRIsure, using an IKA T10 basic Homogenizer (Wilmington, NC, USA). After chloroform treatment and centrifugation, the upper layer was used for total RNA extraction. RNA was precipitated by adding 0.5 mL chilled iso-propanol (100%) followed by incubation for 10 minutes at room temperature. The precipitated RNA solution was centrifuged at 12000× *g* for 10 minutes at 4 °C to obtain an RNA pellet. The pellet was washed by adding 1 mL ethanol (75%) and centrifuging at 7500× *g* for 5 minutes at 4 °C. The resulting RNA pellet was dissolved in 100 µL DEPC-treated water and proceeded to the RNA purification step using an RNeasy Mini Kit (Qiagen, GmbH, Hilden, Germany) as per the manufacturer’s instructions. The elution step for RNA from the spin columns with 50 μL of RNase-free water was repeated, and both elutions were mixed together thoroughly. The quantity and purity of purified total RNA were determined using the spectrophotometer NANODROP-8000 (ThermoFisher Scientific, Wilmington, DE, USA). From the original concentration of RNA, 100-fold (1:100) serial dilutions were prepared and stored at −80 °C until further processing. RNA integrity was examined in Agilent 2100 Bioanalyzer (Agilent Technologies, Waldbronn, Germany) using Agilent RNA 6000 Nano Kit as per the manufacturer’s instructions. The samples with RNA integrity number (RIN) > 8.5 were regarded as high integrity and then used in the downstream qPCR assay.

The middle and bottom layers from the homogenized and chloroform separated samples were mixed and processed for total DNA extraction. Briefly, 0.3 mL absolute ethanol was added to precipitate DNA and samples incubated for 3 minutes at room temperature. The precipitated DNA was centrifuged at 2000× *g* for 5 minutes at 4 °C to obtain a DNA pellet. The pellet was washed with 1 mL of 0.8 M sodium citrate in 10% ethanol and the process was repeated for a total of three times. Next, the pellet was washed with 1.5 mL ethanol (75%) and centrifuged as described earlier. After ethanol removal, the pellet was dissolved in 100 µL TE buffer (10 mM Tris-HCl, 1 mM disodium EDTA, pH 8.0) and centrifuged at 12000× *g* for 10 minutes at 4 °C to remove any insoluble material. The quantity and purity of total DNA in 2 µL of each sample was determined in a NANODROP-8000 spectrophotometer as described earlier. The pure extracted DNA was stored at −20 °C until used for downstream applications.

### Primer design for oligonucleotides

**Figure 4:**
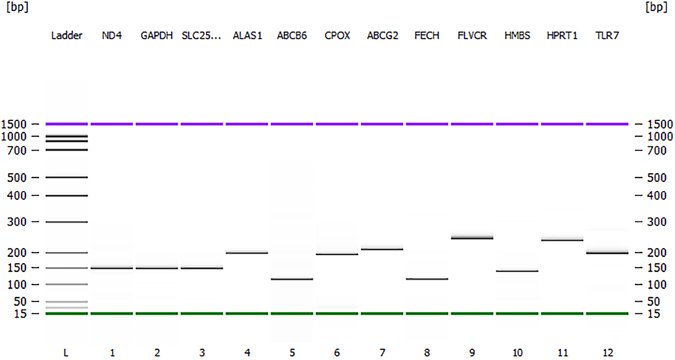
Amplification of the genes fragments from the shell gland tissue of a chicken to assess the specificities of the primers used in the current study. L. DNA marker; (1) *ND4* (137 bp); (2) *GAPDH* (137 bp); (3) *SLC25A38* (139 bp); (4) *ALAS1* (197 bp); (5) *ABCB6* (107 bp); (6) *CPOX* (187 bp); (7) *ABCG2* (208 bp); (8) *FECH* (112 bp); (9). *FLVCR1* (239 bp); (10) *HMBS* (131 bp); (11) *HPRT1* (245 bp); (12) TLR7-positive control (200 bp). The upper (purple) and lower (green) markers act as internal standards and are used to align the ladder analysis with the individual DNA sample analysis. The standard curve (plotting migration time against DNA amplicon size), in conjunction with the markers, is then used to calculate DNA fragment sizes for each well from the migration times measured (see Agilent 2100 Bioanalyzer Users Guide for Molecular Assays). The DNA gel in Agilent 2100 Bioanalyzer was performed as per the manufacturer’s instructions of Agilent DNA 1000 Kit.

The primer sequences were either referenced from literature or designed using NCBI primer and BLAST options (Table 5). Primer quality was checked in “Beacon Designer” software (http://www.premierbiosoft.com/qOligo/Oligo.jsp?PID=1) for possible self and cross secondary structures. To check the sequence specificity, primers were aligned against the NCBI database using BLASTN, Ensemble Chicken Galgal4 and UCSC’s Chicken (*Gallus gallus*) Genome Browser Gateway. Primers quality for single peak generation during melting curve analysis was also checked in uMelt^SM^ web-based online tool. uMelt^SM^ is used for prediction of DNA melting curves and denaturation profiles of PCR products^68^. Prior to real-time qPCR, the primer amplification efficiency and specificity for each primer pair were analysed by using five 10-fold serial dilutions of the purified total DNA/RNA to construct a standard curve. The amplification efficiency was calculated by the equation^69^: E = 10^(1/sl^°^pe)^ − 1. The primers were synthesized by Invitrogen^®^ (ThermoFisher, Australia). Only the primers specifically amplifying the genes of interest were used in the experiment; those with non-specific amplifications were removed from the study. For example, the primers^63^ designed for the *ABCB6* gene were removed due to their amplification of the *UB3* gene. The specificity of the selected primers in amplifications is shown in Fig. 4. The primers amplified single bands with expected sizes according to their designs based on the sequences of the genes. Furthermore, a single peak of melting curves of the primers was also confirmed following real-time PCR amplification (Supplementary Figure 1).

**Table 5:** Forward (F) and reverse (R) sequences of the primers used in the study.

Gene name	Gene symbol	Primer sequence (5′–3′)	Amplicon size (bp)	Annealing temperature °C	Amplification efficiency (%)	Accession No.	Reference
NADH dehydrogenase subunit 4	*ND4* ^a^	F: CGCAGGCTCCATACTACTCG	137	60	94	NC_001323.1	this study
		R: TTAGGGCACCTCATAGGGCT					
Glyceraldehyde-3-phosphate dehydrogenase	*GAPDH* ^b^	F: GGTCACCAAGAAGGTGGAGA	137	63	97	NC_006088.3	this study
		R: GACAGTGCCCTTGAAGTGTC					
Solute carrier family 25, member 38	*SLC25A38*	F: AGACACGGTATGAGAGTGGA	139	63	95	XM_418818.3	this study
		R: ATCCCAGAGAAAGGTGCGTC					
delta-aminolevulinic acid synthase 1	*ALAS1*	F: GGTGGACAGGAAAGGTAAAGA	197	60	98	NM_001018012.1	Li *et al*.^63^
		R: ACTGGTCATACTGGAAGGTG					
ATP binding cassette subfamily C member 6	*ABCB6*	F: CTCAACTGGTTCGGCACCTA	107	60	94	XM_015290086.1	this study
		R: TTCACTGCATCCTTCACCTCC					
Coproporphyrinogen oxidase	*CPOX*	F: GAGAGGACGGTATGTGGAGT	187	60	95	XM_004938236.1	Li *et al*.^63^
		R: TTTGGGATTGCGGAGAAC					
ATP binding cassette subfamily G member 2	*ABCG2*	F: CCTCCTTGTAAACCTCCCTT	208	65	98	XM_421638.4	Li *et al*.^63^
		R: GTAATCTTCACCAGAGCACCTT					
Ferrochelatase	*FECH*	F: TGCTTTGCCGATCACAT	112	60	96	U68033.1	Li *et al*.^63^
		R: CACGGTTCACCACAGACAT					
Feline leukemia virus subgroup C cellular receptor 1	*FLVCR1*	F: CGGGAGTCGTGTTTGAAGAGA	239	63	99	XM_419425.5	this study
		R: CCCAGCGTTCACTTCTTCTCC					
Hydroxymethylbilane synthase	*HMBS*	F: GGCTGGGAGAATCGCATAGG	131	60	94	XM_417846.2	Yin *et al*.^80^
		R: TCCTGCAGGGCAGATACCAT					
Hypoxanthine phosphoribosyltransferase 1	*HPRT1*	F: ACTGGCTGCTTCTTGTG	245	63	98	NM_204848.1	Yang *et al*.^81^
		R: GGTTGGGTTGTGCTGTT					

^a^Gene was used to amplify fragment of mtDNA; ^b^Gene was used to amplify fragment of gDNA.

### Mitochondrial DNA quantification

#### DNA cloning

**Figure 5:**
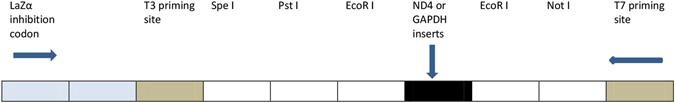
Schematic diagram of single-insert plasmid pCR^TM^4-TOPO (3956 nucleotides). The diagram shows site into which specific fragments of chicken *ND4* or *GAPDH* genes were inserted. The fragments in the study were flanked by restriction and priming sites.

In order to quantify the copy number of mtDNA, a recombinant plasmid vector was constructed by cloning 137 bp fragments of each of the *ND4* and *GAPDH* genes (Table 5), using TOPO^®^ TA Cloning^®^ Kit for sequencing (ThermoFisher Scientific, Australia) as per the manufacturer’s protocol (Fig. 5). The recombinant plasmid was transfected into *Escherichia coli* that was grown overnight on Difco^TM^ LB Agar (Bacto Laboratories, Australia) and then a single colony was enriched in LB broth. Recombinant plasmid DNA from overnight cultured *Escherichia coli* in LB broth was extracted using the PureLink^®^ Quick Plasmid Miniprep Kit (ThermoFisher Scientific, Australia). The procedure was as per the manufacturer’s protocol with 75 µL pre-heated (65 °C) TE buffer used for elution. The eluted recombinant plasmid DNA quality and quantity were checked with the NANODROP-8000 spectrophotometer and stored at −20 °C until used for downstream applications.

#### Standard curve construction

qPCR was performed on the recombinant plasmid DNA at six different dilutions (10^−2^~10^−6^) in 20 µL reaction to check the cloned chicken mtDNA and gDNA amplification efficiencies alongside plasmid DNA amplification. The master-mix preparation and cycling conditions were as per the protocol of the SensiFAST™ SYBR^®^ No-ROX Kit (Bioline, Australia). To construct a standard curve for analysis, eight 10-fold serial dilutions (10^−2^~10^−9^) were prepared from the recombinant plasmid DNA, and qPCR was performed on all the samples as described earlier. The standard curve was constructed by plotting the quantification cycles (Cq) against log_10_ copy numbers of plasmids. All recombinant plasmid constructs were analysed by qPCR and sequencing (AGRF, Australia)^70^ to confirm that accurate cloning of target DNA fragments was executed.

#### Mitochondria DNA copy number quantification by qPCR

Mitochondria were enumerated by the quantification of their DNA copies in a cell and genomic DNA copies were used to represent cell numbers in the samples^71^. Quantitative PCR to quantify mitochondrial DNA copy numbers in a cell was performed with SYBR green method by using the SensiFAST™ SYBR^®^ No-ROX Kit. qPCR reaction was performed in a total volume of 20 µL with a Rotor-Gene 6000 thermocycler (Corbett Research, Sydney, Australia). The reaction consisted of 10 µL 2× SensiFAST™ SYBR^®^ No-ROX mix, 400 nMoles primers, 6.4 µL RNase-free water and 2 µL of diluted DNA (10^−2^ dilution of extracted DNA samples). Reaction without DNA template was included as a no template control (NTC). Recombinant plasmid DNA dilutions were included in the qPCR runs. qPCR was performed following a 2-step protocol: polymerase activation and DNA denaturation at 95 °C for 3 minutes, and 40 cycles of denaturation at 95 °C for 5 seconds and annealing and extension at 60 °C or 63 °C for 30 seconds. Fluorescent signals were acquired at the end of each annealing/extension step during qPCR cycles (40). A melting phase at a ramp from 50 °C to 99 °C at 1°C increment was conducted to assess the specificity of qPCR amplification. The qPCR products were examined in the Bioanalyzer using Agilent DNA 1000 Kit to determine the amplification specificity by the size of the amplicons estimated. qPCR amplification efficiency was determined as previously described. The qPCR data for the genes were processed further when the qPCR amplification efficiency was in a range of 94% to 105%, and linear correlation coefficient R^2^ > 0.980 as these were considered of high standard^72^.

#### Calculation of mitochondria per cell

The cloned plasmid DNA with *ND4* and *GAPDH* genes inserts were converted into plasmid DNA copies/µL in six different dilutions for analysis. Plasmid copy number was calculated based on the concentration of plasmid DNA and its molecular weight. The cloned plasmid DNA amplification cycle (Cq) values were then used to construct a standard curve to calculate the mtDNA and gDNA copies per diploid cell. The absolute copy number of mtDNA per cell was calculated according to the equation: (copy number of mtDNA)/(copy number of gDNA/2).

### Gene expression analysis by qPCR

For the gene expression studies, samples were run in triplicate with the inclusion of NTC and no reverse transcriptase (−RT) controls. Master-mix was prepared as per the manufacturer’s protocol using the SensiFAST SYBR^®^ Lo-ROX One-Step RT-PCR Kit (Bioline, Australia). The reaction in a volume of 20 µL contained 10 µL of 2× SensiFAST SYBR low Rox one-step mix, 400 nMoles primers, 0.2 µL of reverse transcriptase, 0.4 µL of RiboSafe RNase inhibitor, 3.8 µL RNase-free water and 4 µL of RNA template (diluted 100 times). Reverse transcription and amplification were conducted following the thermal cycling protocol in Rotor-Gene 6000: reverse transcription at 45 °C for 10 minutes, polymerase activation and denaturation at 95 °C for 2 minutes, then 40 cycles of denaturation at 95 °C for 5 seconds and annealing and extension at 60 °C, 63 °C or 65 °C for 20 seconds. The fluorescent data collection and melting analysis were performed as described previously.

### Measurement of protoporphyrin IX in tissue and eggshell

The shell gland that was opened for tissue collection for RNA and DNA extractions was used for protoporphyrin IX (PP IX) measurements. From the right half of the shell gland, a 0.25 g piece (including muscle layers) was weighed with an analytical weighing balance (Quintix513-1 S, Göttingen, Germany) and directly transferred into a 10 mL tube containing 4 mL of 3N HCl^73^. The tissue was homogenized (IKA T10 homogenizer) and stored in a refrigerator for 3 hrs. The homogenised tissues were centrifuged at 800× *g* for 30 minutes at 4 °C and the absorbance of the supernatant was read in a spectrophotometer (UV-1201, Shimadzu, Kyoto, Japan) at PP IX specific absorbance wavelength of 412 nm. Standard dilutions (0 to 6.87 nMoles) prepared from protoporphyrin IX di-sodium salt (Sigma Aldrich, Australia) were read at the same wavelength in order to construct a standard curve for the calculations of the amount of PP IX per gram of tissue. The quantification of PP IX in the eggshells was performed according to Samiullah and Roberts^15^.

### Statistical analysis

In order to determine the mitochondrial count, the mtDNA copies per cell data were analysed in SPSS version 22 (IBM Corporation, Armonk, NY, USA)^74^ with the GLM module by taking the time-points and nicarbazin treatment as main effects. Level of significance was indicated by a probability of less than 5%. The Tukey test was used to differentiate levels of significance between mean values.

For the gene expression studies, raw Cq values for all candidate target genes were imported into qbase + version 3.0 (Biogazelle, Belgium)^75^ and analysed against the two reference genes *HMBS* and *HPRT1*, validated in the current study. The qbase + applies an arithmetic mean method to transform logarithmic Cq value to linear relative quantity using exponential function for relative quantification of genes and the arithmetic mean of average Cq is scaled to a given target sample^76, 77^. For data analysis, the basic calculation used in qbase + was based on 2^−ΔΔCq^ method^78^ as the amplification efficiencies of the genes were very close to 100%. In qbase + algorithm, the relative quantity of gene expression level between different samples for a given target gene does not have an absolute scale and is only meaningful in comparison to values obtained for other samples measured for that gene in the same experiment^75^. The result table shows normalized relative quantities (NRQ) values that are calculated across all unknown samples per target gene. The gene expression levels are typically log-normally distributed and all statistical calculations are performed on the log_10_ transformed NRQ values^75, 79^. For easy interpretation of the statistical results, values are re-transformed to linear scale by taking the antilogarithm. Thus, in the current study, the relative expression values for a given gene show fold changes relative to the average expression level across all samples for that particular gene.

To determine the effect of time-points and nicarbazin treatment on expression stability of individual target genes, the NRQ (linear scale) were exported and analysed in SPSS using GLM module. Level of significance was calculated as stated previously. All other parameters data were analysed in SPSS using GLM module unless stated.

## Electronic supplementary material

Melting curves of the amplicons from candidate target genes show that the amplifications were specific
