# Supplementary material for: Downregulation of ALAS1 by nicarbazin treatment underlies the reduced synthesis of protoporphyrin IX in shell gland of laying hens
Source: Sci Rep. 2017 Jul 24;7:6253. doi: 10.1038/s41598-017-06527-y (PMC5524794; doi:10.1038/s41598-017-06527-y)
Supplement: Supplementary file 1 — Melting curves of the amplicons from candidate target genes show that the amplifications were specific [file 41598_2017_6527_MOESM1_ESM.pdf]

Downregulation of *ALAS1* by nicarbazin treatment underlies the reduced synthesis of protoporphyrin IX in shell gland of laying hens

Sami Samiullah, Juliet Roberts, Shu-Biao Wu

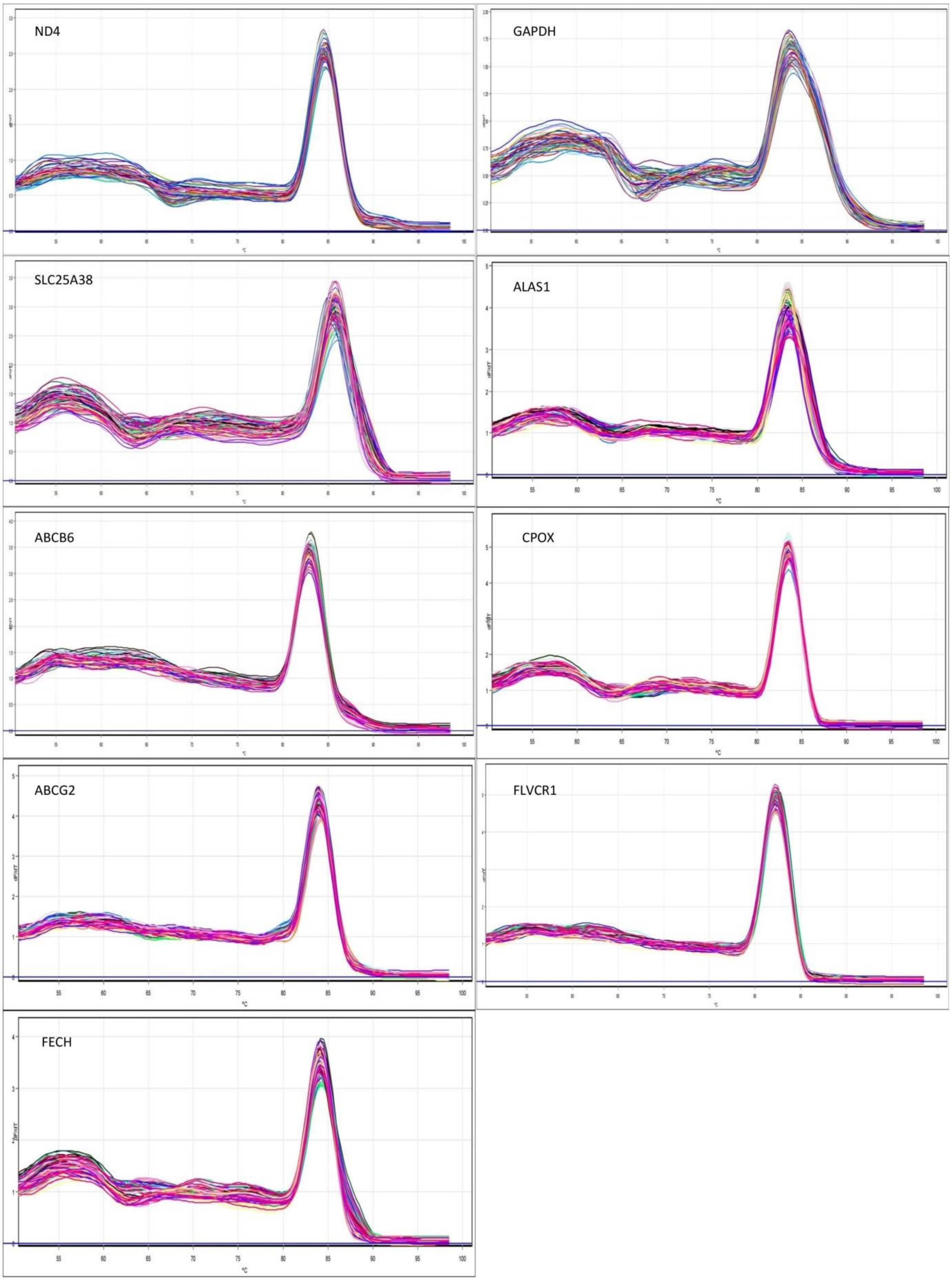

Supplementary Figure 1. Melting curves of the amplicons from candidate target genes show that the amplifications were specific. All of the amplicons showed single peak.

During qPCR, at the end of the amplification, a melting phase at a ramp from 50°C to 99°C at 1°C increment was conducted to assess the specificity of PCR amplification.
